# Coagulation Factor XIII Val34Leu Polymorphism in the Prediction of Premature Cardiovascular Events—The Results of Two Meta-Analyses

**DOI:** 10.3390/jcm11123454

**Published:** 2022-06-15

**Authors:** Beata Sarecka-Hujar, Danuta Łoboda, Elżbieta Paradowska-Nowakowska, Krzysztof S. Gołba

**Affiliations:** 1 Department of Basic Biomedical Science, Faculty of Pharmaceutical Sciences in Sosnowiec, Medical University of Silesia, 41-200 Sosnowiec, Poland; 2 Department of Electrocardiology and Heart Failure, Faculty of Health Sciences in Katowice, Medical University of Silesia, 40-635 Katowice, Poland; dana.loboda@gmail.com (D.Ł.); kgolba@sum.edu.pl (K.S.G.); 3 Department of Electrocardiology, Upper-Silesian Medical Centre, 40-635 Katowice, Poland; 4 Department of Cardiac Rehabilitation, “Ustron” Health Resort, 43-450 Ustroń, Poland; elamed@poczta.fm

**Keywords:** arterial ischemic stroke, myocardial infarction, young adults, FXIII polymorphism

## Abstract

Background: Polymorphisms within the gene that encodes for coagulation factor XIII (FXIII) have been suggested to be involved in the pathogeneses of ischemic stroke (IS) and myocardial infarction (MI). The Val34Leu polymorphism is one of the most commonly analysed *FXIII* polymorphisms. However, studies on the role of the Val34Leu polymorphism in the aetiology of vascular diseases often show contradictory results. In the present meta-analysis, we aimed to pool data from available articles to assess the relationship between the *FXIII* Val34Leu polymorphism and the susceptibilities to IS of undetermined source and premature MI in patients aged below 55 years. Methods: We searched databases (PubMed, Embase, Google Scholar, SciELO, and Medline) using specific keywords (the last search was in January 2022). Eventually, 18 studies (627 cases and 1639 controls for IS; 2595 cases and 4255 controls for MI) met the inclusion criteria. Data were analysed using RevMan 5.4 and StatsDirect 3 link software. The relation between Val34Leu polymorphism and disease was analysed in five genetic models, i.e., dominant, recessive, additive, heterozygous, and allelic. Results: No relation between Val34Leu polymorphism and IS in young adults was observed in all analysed genetic models. For premature MI, significant pooled OR was found between the carrier state of the Leu allele (Val/Leu + Leu/Leu vs. Val/Val) and a lack of MI, suggesting its protective role (OR = 0.80 95%CI 0.64–0.99, *p* = 0.04). A similar finding was observed for the heterozygous model in MI (Val/Leu vs. Val/Val) (OR = 0.77 95%CI 0.61–0.98, *p* = 0.03). No relation was found for the recessive, additive, and allelic models in MI. Conclusions: In the population of young adults, no positive correlation was found between the *FXIII* Val34Leu polymorphism and IS of undetermined source in any of the analysed genetic models. In turn, the carrier state of the 34Leu allele as well as *FXIII* heterozygotes themselves were found to play a protective role in relation to premature MI.

## 1. Introduction

The contribution of both genetic predisposition and well-known conventional risk factors to the pathogenesis of the first cardiovascular (CV) event in young people appears to be obvious.

Factor XIII (FXIII) is a multifunctional pro-γ-transglutaminase involved in the formation and stabilisation of a fibrin clot as well as the aggregation and adhesion of platelets [1,2]. Previously, it was suggested that FXIII may act as a modulator of various cell processes, i.e., migration, adhesion, proliferation, and apoptosis [3,4]. The cellular regulation mediated by active FXIII affects, inter alia, monocytes/macrophages, endothelial cells, and platelets, which have an impact on inflammatory and atherosclerotic processes in the vascular wall [3,4,5].

The common *FXIII* gene polymorphism, G > T transition in the second exon of the *F13A1* gene, results in the substitutive exchange of leucine (Leu) for valine (Val) in the A subunit. Research has found higher activity of FXIII in Leu carriers, while Val homozygotes present a decrease in the activity of this factor [6,7]. The Val34Leu polymorphism of *FXIII* also influences the structure of a fibrin clot, especially in the presence of increased concentrations of fibrinogen, making it much tighter [8]. The role of hemostatic gene variants, e.g., polymorphisms within the gene encoding for coagulation factor XIII, remains of interest in the pathogeneses of ischemic stroke (IS) of undetermined source and premature myocardial infarction (MI) [9,10,11,12,13].

Cryptogenic stroke (CS), possibly embolic, is a common type of IS in the young population, accounting for up to 60% of stroke cases before age 45 and approximately 25% in the 45–49 age range [14]. Its non-atherosclerotic origin (embolic stroke of undetermined source, or ESUS) is supported by the lack of cardiometabolic risk factors or atherosclerotic changes in large proximal arteries and the non-lacunar location of infarcts on neuroimaging found in 65% of cases [15,16]. Undiagnosed episodes of atrial fibrillation with secondary thrombus formation in the left atrial appendage account for up to 30% of IS in the elderly [17] and may result in an ischemic event in the younger population as well. However, in younger people, deep venous thrombosis, including asymptomatic presentations, contributes to CS/ESUS pathogenesis in 10–22% of cases in the presence of cardiac right-to-left shunts, such as patent foramen ovale (PFO) [18]. Accordingly, the prevalence of inherited or acquired hypercoagulable states ranges from 3% to 21% in this type of stroke in groups <50 years old [19,20]. In addition, hypercoagulable conditions that cause arterial thrombosis, including antiphospholipid syndrome, can lead to cerebral embolism from intracardiac sources or cause in situ thrombosis in the cerebral, carotid, and coronary arteries without pre-existent significant stenosis [21].

In turn, the most common cause of premature MI or IS in people with unfavourable family history and/or conventional atherogenic risk factors is accelerated atherogenesis and atherothrombosis [22]. Atherosclerotic changes appear in the early decades of life (in the coronary vessels in the second decade and the cerebral vessels in the third decade) due to an inflammatory process in the arterial vascular wall. Plaque build-up is secondary to endothelial dysfunction, the proliferation of smooth muscle cells, the synthesis of connective tissue matrix, and the active accumulation of macrophages and lipids under the influence of inflammatory cytokines [23]. The rupture or erosion of unstable atherosclerotic plaque results in an acute thrombotic event [24]. Polymorphic variants of genes related to lipid metabolism, coagulation cascade, the renin–angiotensin pathway, or endothelial nitric oxide synthesis are common in the general population and increase the likelihood of atherosclerotic CV disease [25].

The aim of the present study was to summarise the results of available data regarding the relationship between *FXIII* Val34Leu polymorphism and premature CV events of atherosclerotic or thrombotic origin in the population of young adults.

## 2. Materials and Methods

### 2.1. Search Strategy

We searched five databases (PubMed, MEDLINE, Embase, SciELO, and Google Scholar) to identify available data published before January 2022 with the use of appropriate keywords: (“FXIII polymorphism” or “factor XIII polymorphism” or “Val34Leu polymorphism”) and (“ischemic stroke” or “stroke” or “myocardial infarction”) and (“young adults” or “young” or “premature” or “early”). The identified studies were included in accordance with the population, intervention, comparison, and outcome (PICO) model to select the relevant research question: Is *FXIII* Val34Leu polymorphism related to IS and MI susceptibility when comparing the prevalence of its alleles and genotypes in young patients with IS and MI against that in controls, according to five genetic models?

### 2.2. Inclusion/Exclusion Criteria for Analysed Studies

Searched studies were included in the meta-analysis if: (a) there was confirmed ischemic stroke or myocardial infarction, (b) a case–control study methodology was used, (c) the age of the patients was below 55 years, (d) access to genotypes distribution was available, (e) the article was a full-length paper or brief communication, and (f) the article was written in English. Studies were excluded from the meta-analysis for the following reasons: (a) unavailability of genotyping results, (b) lack of reference (control) group, (c) lack of information on the age of the patients, or if the age of the patients was above 55 years, (d) if the material took the form of conference proceedings, review articles, case reports, or meta-analyses, or if the study was an animal study, and (e) if the article was written in a language other than English. When subgroups of patients younger than age 55 were available in the included studies, we used the data regarding those patients.

**Figure 1:**
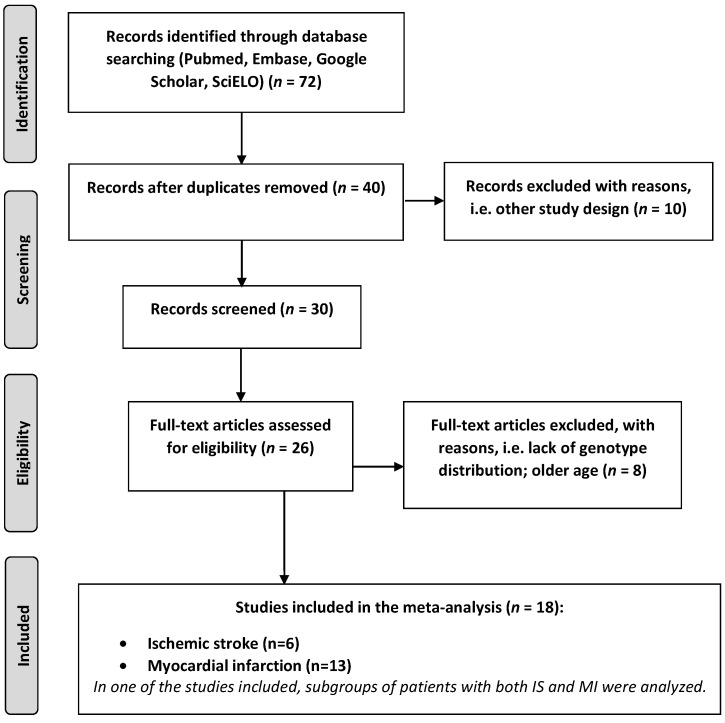
Flow chart presenting the process of searching for eligible articles.

Finally, 18 case–control studies on young adults analysing *FXIII* polymorphism with regards to IS and MI met the inclusion criteria (3222 cases and 5894 controls in total), including 6 studies on IS (627 cases with stroke and 1639 controls [9,11,12,26,27,28]) and 13 studies on MI (2595 cases with premature MI and 4255 controls [10,12,13,29,30,31,32,33,34,35,36,37,38]). In the study by Reiner et al. [12], subgroups of patients with both IS and MI were analysed. Figure 1 displays the flow diagram of the search process and reasons for excluding the studies.

### 2.3. Data Extraction and Methodological Quality

From each study that was included, the following data were extracted: the first author’s name, the year of the publication, the number of cases and controls, the ages of the cases and control subjects, and the number of the particular genotypes of FXIII Val34Leu polymorphisms in both patients and controls. Allele frequencies were calculated based on genotype frequencies. Additionally, we used the Hardy–Weinberg equilibrium (HWE) to check the consistency of genotype distribution at the significance level of 0.05 for controls in each study that was included. The Newcastle–Ottawa scale (NOS) for case–control studies was used to evaluate the methodological quality of the included studies [39]. Using the NOS scale, points from 0 to 9 were assigned to each study. A study was considered to be of sufficient quality when the article achieved at least five points. In the case of deviation from HWE, the assumption of Minelli et al. [40] was adopted (to not exclude these studies when no other grounds for doubting the quality of the study were present).

### 2.4. Statistical Analyses

Statistical analyses were conducted twice using the Review Manager software (RevMan version 5.4; Cochrane, London, UK) and StatsDirect 3 link software (version 3.3.5; StatsDirect Ltd., Wirral, UK). We calculated the pooled odds ratio (OR) with a 95% confidence interval (CI) to determine the strength of association between the particular genetic model and the disease, that is, IS or MI. We selected the statistical model of the analyses (random or fixed) based on heterogeneity between the included studies; this was assessed using the I^2^ test, which describes the proportion of variance (from 0% to 100%) due to variance in true effect sizes rather than sampling error. I^2^ values of 25%, 50%, and 75% were correlated with low, intermediate, and high inconsistency, respectively. The random effects method (DerSimonian–Laird; REM) was used to calculate the pooled OR with a 95% CI when heterogeneity between the studies was significant; otherwise, the calculation was performed with the fixed-effects method (Mantel–Haenszel; FEM). The strength of the correlation between the FXIII Val34Leu polymorphism and IS and MI was assessed in the following models: dominant (Val/Leu + Leu/Leu vs. Val/Val), recessive (Leu/Leu vs. Val/Val + Val/Leu), additive (Leu/Leu vs. Val/Val), heterozygous (Val/Leu vs. Val/Val), and allelic (Leu vs. Val). To evaluate the stability of the results, sensitivity analyses were made via the sequential exclusion of each study.

To assess potential publication bias, both Egger’s regression and Begg’s rank correlation tests were performed. The result was considered statistically significant if the *p* value was below 0.05.

## 3. Results

### 3.1. Ischemic Stroke

#### 3.1.1. Characteristics of the Studies Included

Characteristics of the six included studies analysing Val34Leu polymorphism within the *FXIII* gene and ischemic stroke in young patients are shown in Table 1. The genotype frequencies in control subjects were in agreement with HWE in all included studies. The dominant genotyping method was PCR-RFLP (in three out of six studies) [10,12,27]. The largest groups of both patients and controls were analysed by Pruissen et al. [26] and Shemirani et al. [28], whereas the fewest patients were recruited by Reiner et al. [12] and Ranellou et al. [27]. From the study by Shemirani et al. [28], a subgroup of female patients was extracted since the whole study group was much older and above the age we assumed as an inclusion criterion.

#### 3.1.2. Association between FXIII Val34Leu Polymorphism and IS in Young Patients

**Figure 2:**
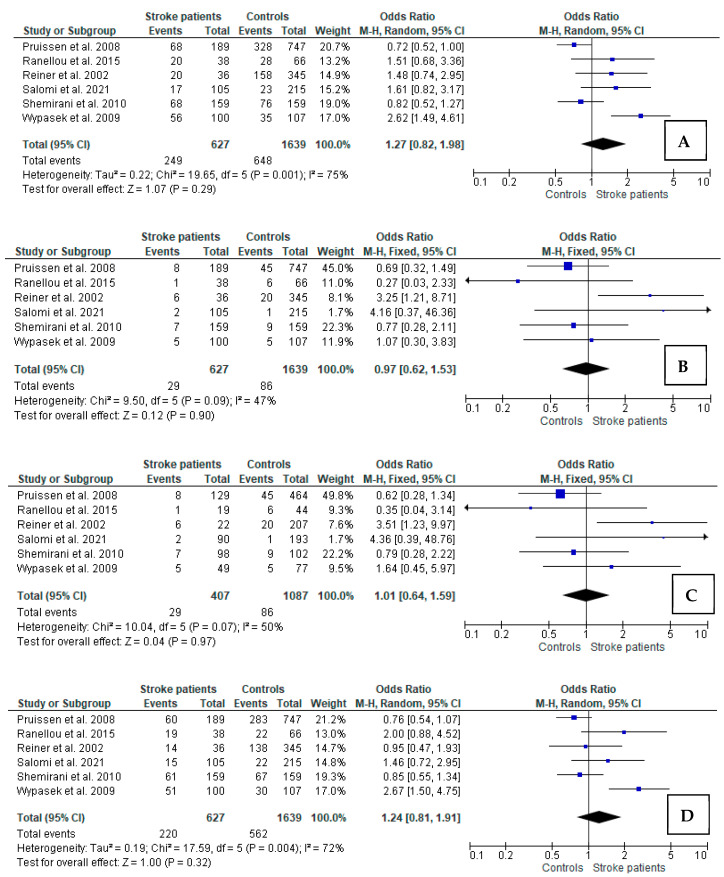

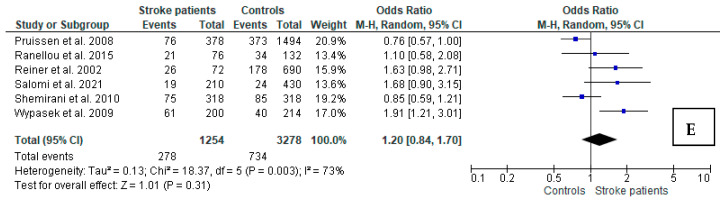
Forest plots for relations between different genetic models of FXIII polymorphism and ischemic stroke in total groups of young patients: (**A**) Val/Leu + Leu/Leu vs. Val/Val; (**B**) Leu/Leu vs. Val/Leu + Val/Val; (**C**) Leu/Leu vs. Val/Val; (**D**) Val/Leu vs. Val/Val; (**E**) Leu vs. Val. M-H: Mantel–Haenszel; CI: confidence interval; I^2^: heterogeneity; df: degrees of freedom [10,11,12,26,27,28].

Significant heterogeneity was observed for the dominant, heterozygous, and allelic analyses; thus, REM was used to calculate pooled OR. For recessive and additive models, no heterogeneity between the included studies was found; thus, pooled OR was assessed with FEM. In the case of the dominant model analysis of *FXIII* polymorphism (Val/Leu + Leu/Leu vs. Val/Val), no relation between the carrier state of the Leu allele and IS in young adults was observed. Similar findings were observed for the recessive (Leu/Leu vs. Val/Val + Val/Leu), additive (Leu/Leu vs. Val/Val), and heterozygous model (Val/Leu vs. Val/Val), as well as for the allelic (Leu allele vs. Val allele) models (Figure 2).

#### 3.1.3. Sensitivity Analyses

In the sensitivity analysis, no change in the OR value was demonstrated in the case of all analysed genetic models after excluding subsequent studies. Therefore, these analyses were considered stable.

#### 3.1.4. Publication Bias in the Total Group of Studies Analysing Val34Leu Polymorphism in the *FXIII* Gene and IS in Young Patients

Regarding the analyses for IS, publication bias was observed for the dominant and heterozygous genetic models. For the remaining models, no publication bias was observed since the shapes of the funnel plots were roughly symmetrical. Table 2 shows the exact results of both Egger’s and Begg’s tests for all genetic models between stroke patients and controls.

### 3.2. Myocardial Infarction

#### 3.2.1. Characteristics of the Studies

Characteristics of the thirteen studies analysing Val34Leu polymorphism within the *FXIII* gene and premature MI are shown in Table 3. The genotype frequencies in control subjects agreed with HWE in all included studies except for the study by Hancer et al. [33]. In most included studies, the PCR-RFLP method was used to genotype the *FXIII* Val34Leu polymorphism (in 7 out of 13 studies) [10,12,30,31,32,33]. The largest groups of both patients and controls were analysed by the Atherosclerosis, Thrombosis, and Vascular Biology Italian Study Group [31]; Silvain et al. [10]; and Siegerink et al. [37], whereas the fewest young patients were analysed by Alkhiary et al. [29], Butt et al. [32], and Roldan et al. [36].

#### 3.2.2. Association between *FXIII* Val34Leu Polymorphism and MI in Young Patients

**Figure 3:**
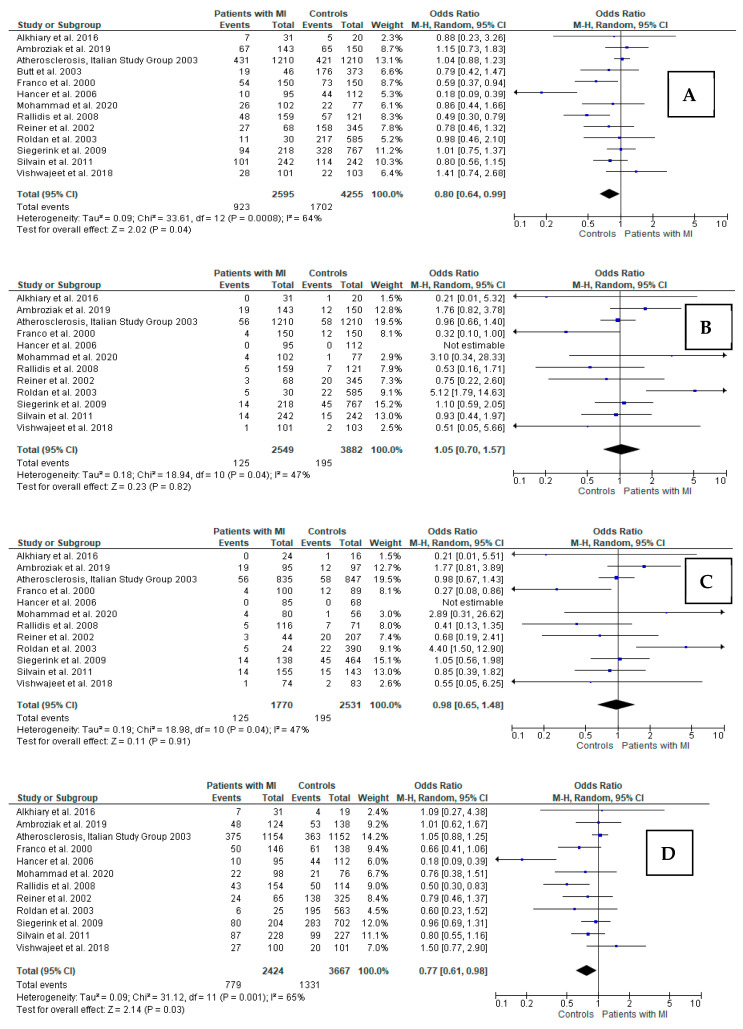

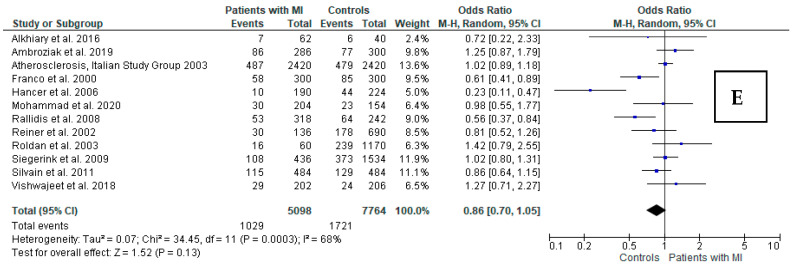
Forest plots for relations between different genetic models of FXIII polymorphism and myocardial infarction in total groups of young patients: (**A**) Val/Leu + Leu/Leu vs. Val/Val; (**B**) Leu/Leu vs. Val/Leu + Val/Val; (**C**) Leu/Leu vs. Val/Val; (**D**) Val/Leu vs. Val/Val; (**E**) Leu vs. Val. M-H: Mantel–Haenszel; CI: confidence interval; I^2^: heterogeneity; df: degrees of freedom [10,12,13,29,30,31,32,33,34,35,36,37,38].

Significant heterogeneity was observed in all genetic models; thus, REM was used to calculate pooled OR. In the case of dominant model analysis of *FXIII* polymorphism (Val/Leu + Leu/Leu vs. Val/Val), significant pooled OR was demonstrated between the carrier state of the Leu allele and a lack of MI, suggesting its protective role (OR = 0.80 95%CI 0.64–0.99, *p* = 0.04). A similar finding was observed for the heterozygous model (Val/Leu vs. Val/Val; OR = 0.77 95%CI 0.61–0.98, *p* = 0.03; Figure 3). No relation was found for the recessive, additive, and allelic models.

#### 3.2.3. Sensitivity Analyses

During sensitivity analysis, no change in the OR value was demonstrated in the cases of the recessive, additive, and allelic genetic models for MI after excluding subsequent studies. Therefore, these analyses were considered stable. However, in the case of the dominant model, after excluding subsequent studies by Butt et al. [32], Franco et al. [13], Hancer et al. [33], Rallidis et al. [35], Reiner et al. [12], and Silvain et al. [10], the significance of the results was lost in REM analysis. Similarly, in the case of the heterozygous model, the results were not significant after omitting the data from the studies by Franco et al. [13], Hancer et al. [33], and Rallidis et al. [35]. Thus, these analyses should be treated with caution.

#### 3.2.4. Publication Bias in the Total Group of Studies Analysing Val34Leu Polymorphism in the *FXIII* Gene and MI in Young Patients

For all of the genetic models, no publication bias was observed since the shapes of the funnel plots were roughly symmetrical. Table 4 shows the exact results of both Egger’s and Begg’s tests for all genetic models between MI patients and controls.

## 4. Discussion

The results of the present meta-analysis show different relationships for two types of ischemic events with different pathogeneses. In the case of IS with a cryptogenic background, often secondary to thromboembolic processes, we observed no relation with *FXIII* Val34Leu polymorphism in each genetic model analysed. On the other hand, when we collected patients with premature MI most often caused by accelerated atherosclerotic processes, we demonstrated that carrying the 34Leu allele (i.e., Val/Leu or Leu/Leu genotypes) could have a protective role. The carrier state of the Leu allele was more common in controls compared to young patients with MI (40% vs. 35.6%, respectively). Subjects with Val/Leu genotypes were more frequent in controls than in MI patients (36.3% vs. 32.1%, respectively) in reference to wild-type homozygous Val/Val, which may also suggest a protective effect. However, these results should be treated with caution since there was some loss of significance after omitting subsequent studies. The results for the remaining genetic models did not reveal significance between *FXIII* polymorphism and premature MI.

Coagulation factor polymorphisms, including *FXIII* polymorphisms, have been analysed in the context of premature CV events, including coronary artery disease (CAD) [33,41], IS [42], haemorrhagic stroke [43], and venous thromboembolism [44] in various populations and age ranges. Focusing on the young adult population makes it possible to reveal the influence of genetic factors, which in the population aged ≤55 may still prevail over the influence of environmental factors, undiagnosed atrial tachyarrhythmias, and other major classic CV risk factors.

Numerous data confirmed the protective effect of 34Leu allele carriage on the development of premature MI. This effect was stronger in the 18–50-year-old population than in patients over 50 years of age [11,12,33,35,36,45]. Most of the studies based their observations primarily on the higher frequency of the 34Val allele and the lower frequency of the 34Leu allele in the MI groups [13,33,45].

Val34Leu polymorphism is characterised by high ethnic variability. The prevalence of the 34Leu allele in Caucasians has been estimated at 37–51% [12,36,42,44], while it shows lower prevalence among inhabitants of the Middle East (14–37%) [29], South Asia (12%) [38], and the Far East (up to 2.5%) [41,46]. Thus, the comparison of ethnically different groups may give misleading results. It is known that the development of premature MI/IS is influenced by many other genetic and environmental risk factors, including those indirectly related to ethnicity, e.g., the type of diet (protective role of the Mediterranean diet), the percentage of obese people in the population, habit and manner of smoking (pipes, cigars, glass pipes, shishas), the percentage of women using oral contraception, and polymorphisms regarding other genes related to the development of atherothrombosis and hypercoagulability [47,48,49,50,51].

In 1210 young adult Italian people [31] with a history of MI, the effects of major CV risk factors, including family history (OR = 4.0), smoking (OR = 7.6), hypertension (OR = 4.5), being overweight (OR = 1.6), dyslipidaemia (OR = 1.4), and diabetes (OR = 7.4), were greater than the effects of genes involved in clotting, platelet function, fibrinolysis, or homocysteine metabolism, including the *FXIII* 34Leu variant (OR = 1.1). In a group of 1030 Turkish patients, the protective role of the *FXIII* Val34Leu polymorphism against MI was confirmed (OR = 0.31), but it was not an independent variable when major CV risk factors were taken into account in multivariate analysis [33].

Franco et al. [13] reported different results and confirmed that the carrier state of the 34Leu allele reduced the risk of MI related to metabolic risk factors. Individuals who did not carry the 34Leu allele had a 13.9-fold higher risk of MI in the presence of hypertension, diabetes, dyslipidaemia, and obesity, while in 34Leu allele carriers, the risk was reduced to 6.8. In addition, the *FXIII* 34Leu variant significantly reduced the risk of MI among smokers (OR = 3.9 in 34Leu allele carriers vs. OR = 6.1 in non-carriers). In the above study, the risk reduction was greater in homozygotes than in heterozygotes for the Leu allele, suggesting a gene dosage effect.

Importantly, the meta-analysis by Jung et al. [52] found that the Val/Val genotype was associated with CAD in MI only and not in chronic coronary syndrome. Additionally, in a smaller group of Greek patients, the protective effect of the 34Leu allele carrier was limited to those with significant atherosclerotic lesions in the coronary arteries [35]. It cannot be ruled out that increased thrombogenicity has clinical significance and results in the development of CV events, especially in the presence of genetically induced atherosclerotic plaques susceptible to rupture [24]. However, some clinical studies on the involvement of FXIII in inflammatory processes [4,53] may support the hypothesis about the atherogenic influence of this factor’s polymorphisms, alone or in association with other genes, in the development of premature atherosclerotic lesions. The identification of groups with increased CV risk may contribute each time to the earlier introduction of pharmacological prophylaxis, e.g., statins and acetylsalicylic acid, in the primary prevention of atherosclerotic events.

Conversely, most researchers did not confirm the protective effect of the Val34Leu polymorphism in relation to IS [26,27,54,55,56,57] or describe a higher percentage of 34Leu allele carriers in groups of patients who experienced cerebrovascular events, particularly in the presence of a PFO [9,11,12,58].

Elbaz et al. [59] described the protective effect of the 34Leu allele in a group of 456 patients aged 69 (20–85) years with IS (OR 0.58); this effect was independent of traditional CV risk factors and even exceeded the effect of smoking. However, in the population with IS, most researchers found no correlation between age, gender, the presence of traditional CV risk factors, the type of acute cerebrovascular event, and the Val34Leu genotype [11,26,42,54,57,59]. It is worth mentioning that Undas et al. [60] demonstrated higher anti-aggregation effectiveness from a low dose of aspirin in 34Leu allele carriers than in patients with the Val/Val genotype, and that the effect was also more significant for smokers.

An additional problem that makes it difficult to confidently assess the contribution of gene polymorphisms in CS patients is the presence of undetected, asymptomatic atrial tachyarrhythmias such as atrial fibrillation and atrial flutter. Large clinical trials [61,62] have confirmed the effectiveness of long-term ECG monitoring in verifying the causes of IS, with 9–10% of confirmed FA episodes in the ESUS patient groups. However, in these subgroups of young people at low risk, as assessed by the CHA₂DS₂-VASc score (0–1 points), refining the thromboembolic risk by assessing genetic predisposition may lead to earlier initiation of anticoagulant treatment for the primary prevention of IS, regardless of confirmation of atrial fibrillation.

It has also been proven that the interactions between various genetic factors and gene polymorphisms involved in the coagulation and fibrinolysis processes are important in the pathogeneses of premature atherosclerosis and hypercoagulability, and that their synergistic effect may be crucial in the development of CAD/MI [47,63] and IS [64]. Butt et al. [32] reported a 12-fold increase in MI risk in 500 Newfoundland inhabitants in whom the *FXIII* Leu34 allele and prothrombin 20210G > A (*FII* 20210A) coexisted. In turn, a decrease in the risk of MI was found in 34Leu carriers with high fibrinogen levels [45,51]. Reiner et al. [12] reported an increased risk of IS associated with the Leu34/Leu34 genotype, though only among young women who carried the alpha2 807T integrin allele, which was previously described as a risk factor for IS at a young age. It is worth mentioning that in the study by González-Conejero et al. [65], the efficacy and safety of fibrinolytic therapy in acute IS varied depending on the type of Val34Leu polymorphism and fibrinogen concentration. Carriers of the 34Leu variant with a high concentration of fibrinogen (>3.6 g/L) were less responsive to fibrinolysis. Moreover, patients with the 34Leu allele and patients with high fibrinogen concentration had a higher risk of severe haemorrhagic infarction and death following such therapy. These reports are consistent with the results of authors describing the synergistic effect of other hemostatic gene polymorphisms in increasing the risk of MI [47] and IS [66,67].

The present meta-analysis has some limitations. No additional data on other factors that could interact with the analysed *FXIII* polymorphism in the development of CV were available. Meta-analyses of some specific interactions between particular genes and factors that are simultaneously present in patients would be more accurate for understanding the role of the analysed polymorphism and the disease. In the case of *FXIII* Val34Leu polymorphism, the level of factor XIII should be especially considered.

## 5. Conclusions

The assessment of gene polymorphisms involved in the processes of coagulation and fibrinolysis may be important in the primary prevention of cardiovascular events in a group of young adults without classic risk factors. In young adults, no positive correlation was found between the *FXIII* Val34Leu polymorphism and IS in any of the analysed genetic models. In the case of premature MI, our meta-analysis demonstrated that the carrier state of the 34Leu allele might play a protective role in premature MI. In both cases, the influence of gene–gene and gene–environment interactions on disease development should be taken into account.

## Figures and Tables

**Table 1:** Characteristics of the studies included to the meta-analysis regarding the relation between *FXIII Val34Leu* polymorphism and IS in young adults.

Study (year)	Patients with Premature Ischemic Stroke						Controls					Genotyping Method	Indicated Relation	HWE (for Controls) (ꭓ^2^; *p*)	QUALITY (Newcastle-Ottawa Scale)
	Population	Age	N	Genotypes of *FXIII Val34Leu* Polymorphism			Age	N	Genotypes of *FXIII Val34Leu* Polymorphism						
				Val/Val	Val/Leu	Leu/Leu			Val/Val	Val/Leu	Leu/Leu				
Pruissen et al. [26]	Netherlands	Mean age: 39.8 years	189	121	60	8	Mean age: 38.6 years	747	419	283	45	5′nuclease/TaqMan assay	No	0.093; 0.95	8
Ranellou et al. [27]	Greece	Mean age: 37.8 years	38	18	19	1	Mean age: 38 years	66	38	22	6	PCR–RFLP method	No	1.089; 0.58	8
Reiner et al. [12]	USA	Mean age: 37.9 years	36	16	14	6	Mean age: 37.7 years	345	187	138	20	PCR–RFLP method	Yes, between Leu34 homozygotes and IS	0.693; 0.71	8
Salomi et al. [10]	India	Mean range: 32.7 years	105	88	15	2	Mean age: 31.8 years	215	192	22	1	PCR-RFLP method	No	0.183; 0.91	9
Shemirani et al. [28]	Hungary	Median age: 47 years	159	91	61	7	Median age: 47 years	159	83	67	9	Real time PCR	No	0.913; 0.63	9
Wypasek et al. [11]	Poland	Mean age: 43.4 years	100	44	51	5	Mean age 43.6 years	107	72	30	5	Single nucleotide polymorphism (SNP) analysis	Yes	0.644; 0.72	6
**TOTAL**			627	378	220	29	**TOTAL**	1639	1001	562	86				

**Table 2:** The results of Egger’s and Begg’s tests for all genetic models between the studies analysing stroke patients and controls.

Genetic Model	Egger’s Test			Begg’s Test	
	Intercept	95% CI	*p*	Kendall’s Tau	*p*
Dominant	4.543	−0.584 to 9.670	0.070	0.200	0.719
Recessive	0.359	−4.509 to 5.227	0.848	0.200	0.719
Additive	0.975	−3.906 to 5.857	0.609	0.333	0.469
Heterozygous	3.990	−1.174 to 9.154	0.098	0.467	0.272
Allelic	4.508	−0.644 to 9.660	0.072	0.200	0.719

CI: confidence interval.

**Table 3:** Characteristics of the studies included to the meta-analysis regarding relation between *FXIII Val34Leu* polymorphism and myocardial infarction in young adults.

Study (year)	Patients with Premature Myocardial Infarction						Controls					Genotyping Method	Indicated Relation	HWE(for Controls) (χ^2^; p)	QUALITY (Newcastle-Ottawa Scale)
	Population	Age	N	Genotypes of *FXIII Val34Leu* Polymorphism			Age	N	Genotypes of *FXIII Val34Leu* Polymorphism						
				Val/Val	Val/Leu	Leu/Leu			Val/Val	Val/Leu	Leu/Leu				
Alkhiary et al. [29]	Egypt	Mean age: 34.16 ± 3.65 years	31	24	7	0	Mean age: 32.25 ± 3.89 years	20	15	4	1	The CVD Strip Assay method	No	0.930; 0.33	7
Amboziak et al. [30]	Poland	Age < 50 years	143	76	48	19	Age-matched to patients	150	85	53	12	PCR–RFLP method	No	0.822; 0.36	8
Atherosclerosis, Thrombosis, and Vascular Biology Italian Study Group [31]	Italian	Age < 45 years	1210	779	375	56	Age-matched to patients	1210	789	363	58	PCR–RFLP method	No	3.681; 0.06	9
Butt et al. [32]	Canada	Age < 50 years	46	27	19 carriers of 34Leu allele		Age < 50 years	373	197	176 carriers of 34Leu allele		PCR–RFLP method	No		6
Franco et al. [13]	Brazil	Mean range: 43 years	150	96	50	4	Mean age: 42 years	150	77	61	12	PCR–RFLP method	Yes, protective role for carriers of 34Leu allele	0.003; 0.99	8
Hancer et al. [33]	Turkey	Age range: 18–50 years	95	85	10	0	Age range: 18-50 years	112	68	44	0	PCR–RFLP method	Yes, protective role for carriers of 34Leu allele	6.692; **0.01**	9
Mohammad et al. [34]	Iraq	Mean age: 42.4 ± 6.19 years	102	76	22	4	Mean age: 41.6 ± 7.09 years	77	55	21	1	The CVD Strip Assay method	No	0.414; 0.52	8
Rallidis et al. [35]	Greece	Mean age: 32.1 ± 3.6 years	159	111	43	5	Mean age: 31.6 ± 3.8 years	121	64	50	7	The CVD Strip Assay method	Yes, protective role was observed	0.467; 0.49	8
Reiner et al. [12]	USA	Mean age: 39.8 years	68	41	24	3	Mean age: 37.7 years	345	187	138	20	PCR–RFLP method	No	0.693; 0.71	8
Roldan et al. [36]	Spain	Mean age: 44.8 ± 6.7 years	30	19	6	5	Mean age: 47.6 ± 19.8 years	585	368	195	22	PCR– allele-specific restriction assay method	Yes	0.376; 0.54	7
Siegerink et al. [37]	The Netherlands	Mean age: 42.9 ± 6.0 years	218	124	80	14	Mean age: 38.6 ± 8.0 years	767	419	283	45	The 5′ nuclease/TaqMan assay	No	0.093; 0.76	6
Silvain et al. [10]	France	Mean age: 39.1 ± 5.3 years	242	141	87	14	Mean age: 39.1 ± 5.3 years	242	128	99	15	PCR–RFLP method	No	0.519; 0.47	8
Vishwajeet et al. [38]	India	Mean age: 37.1 ± 4.3 years	101	73	27	1	Mean age: 30.6 ± 5.9 years	103	81	20	2	Amplification-created restriction enzyme sitePCR	No	0.332; 0.56	8
**TOTAL**			2595	1672	779	125	**TOTAL**	4255	2533	1331	195				

**Table 4:** The results of Egger’s and Begg’s tests for all genetic models between the studies analysing MI patients and controls.

Genetic Model	Egger’s Test			Begg’s Test	
	Intercept	95% CI	*p*	Kendall’s Tau	*p*
Dominant	−1.462	−3.350 to 0.427	0.116	−0.102	0.590
Recessive	−0.169	−2.058 to 1.719	0.844	−0.164	0.445
Additive	−0.322	−2.203 to 1.559	0.708	−0.200	0.359
Heterozygous	−1.592	−3.517 to 0.334	0.095	−0.242	0.249
Allelic	−1.258	−3.426 to 0.910	0.225	−0.242	0.250

CI: confidence interval.

## Data Availability

The data presented in this study are available on request from the Department of Basic Biomedical Science, Faculty of Pharmaceutical Sciences in Sosnowiec, Medical University of Silesia in Katowice (Poland). The data are not publicly available due to privacy restrictions.
